# CD161^+^CD4^+^ T cells are enriched in the liver during chronic hepatitis and associated with co-secretion of IL-22 and IFN-γ

**DOI:** 10.3389/fimmu.2012.00346

**Published:** 2012-11-20

**Authors:** Yu-Hoi Kang, Bianca Seigel, Bertram Bengsch, Vicki M. Fleming, Eva Billerbeck, Ruth Simmons, Lucy Walker, Chris B. Willberg, Eleanor J. Barnes, Anisha Bhagwanani, Ye H. Oo, Hubert E. Blum, David H. Adams, Robert Thimme, Paul Klenerman

**Affiliations:** ^1^Peter Medawar Building for Pathogen ResearchOxford, UK; ^2^Department of Medicine II, University of FreiburgFreiburg, Germany; ^3^Spemann Graduate School of Biology and Medicine, University of FreiburgFreiburg, Germany; ^4^Institute for Biomedical Research, University of BirminghamBirmingham, UK; ^5^Biomedical Research Centre, John Radcliffe HospitalOxford, UK

**Keywords:** CD4^+^ T cell, IL-22, HCV, hepatic inflammation, CD161

## Abstract

Hepatitis C virus infection is a major cause of chronic liver disease. CD4^+^ T cells play a key role in disease outcome. However, the critical functions and associated phenotypes of intrahepatic CD4^+^ T cells are not well defined. We have previously shown that CD8^+^ T cells expressing the C type lectin CD161 are highly enriched in the human liver, especially during chronic hepatitis. These cells are associated with a type 17 differentiation pattern and express cytokines including IL-17A, IL-22, and IFN-γ. We therefore analyzed expression of CD161 on CD4^+^ T cells in blood and liver and addressed the relevant phenotype and functional capacity of these populations. We observed marked enrichment of CD161^+^CD4^+^ T cells in the liver during chronic hepatitis such that they are the dominant subtype (mean 55% of CD4^+^ T cells). IL-22 and IL-17 secreting CD4^+^ T cells were readily found in the livers of HCV^+^ and NASH donors, although not enriched compared to blood. There was, however, specific enrichment of a novel subset of IL-22/IFN-γ dual secretors (*p* = 0.02) compared to blood, a result reconfirmed with direct *ex vivo* analyses. These data indicate the dominance of CD161^+^ expressing lymphocyte populations within the hepatic infiltrate, associated with a distinct cytokine profile. Given their documented roles as antiviral and hepatoprotective cytokines respectively, the impact of co-secretion of IFN-γ and IL-22 in the liver may be particularly significant.

## INTRODUCTION

Hepatitis C virus (HCV) infects 170 million people worldwide and is a major cause of liver disease (Lauer and Walker, 2001). The role of T cells in control of infection has been extensively studied and it is clear that both CD4^+^ and CD8^+^ T cells contribute importantly to disease outcome. There is evidence from animal models, immunogenetics and correlative studies that both sets of T cells are involved in control of viremia after acute infection (Cooper et al., 1999; Lechner et al., 2000; Thimme et al., 2002; McKiernan et al., 2004; Bowen and Walker, 2005). However, in the majority of those infected, the virus is able to evade innate and adaptive immune responses and to establish persistence. In these individuals, T cell infiltrates are still maintained within liver tissue (Koziel et al., 1992; Wong et al., 1998). However, their subsequent composition and overall roles in control of virus, immune-mediated pathology, and tissue remodeling are not well defined.

In terms of CD4^+^ T helper cell responses, much attention has focused on Th1 cells, since secretion of interferon-gamma (IFN-γ) has been proposed to be linked to control of hepatotropic viruses (Guidotti et al., 1999). Some studies indicated the presence of Th2 responses in chronic infection, which may play a role in outcome (Prezzi et al., 2001; Nelson et al., 2003). Recently some attention has turned to Treg responses, characterized by expression of the transcription factor FOXP3. These are enriched within the liver (Ward et al., 2007) and may play a modifying role by down-regulating immunopathogenic as well as antiviral responses. A further T cell subset has been more recently described, named Th17, or type 17 T cells, associated with IL-17A production (McGeachy and Cua, 2008; Tesmer et al., 2008). IL-17A is a pleiotropic cytokine which has been linked to induction of tissue inflammation and recruitment of inflammatory cells in models of inflammatory bowel disease and encephalomyelitis.

Type 17 cells also produce other cytokines, including, importantly, IL-22. This molecule, which is of the IL-10 family, has been shown to be hepatoprotective in murine models of hepatitis (Wolk and Sabat, 2006; Zenewicz et al., 2007). In such models, depletion of IL-17 had little impact on the course of inflammation, but blockade or knockout of IL-22 had a major deleterious effect. It is thought the cytokine acts through promoting anti-apoptotic pathways or pro-proliferative pathways in IL-22R^+^ hepatocytes (Fickenscher et al., 2002; Pan et al., 2004; Dambacher et al., 2008). A similar activity in skin may promote the pathogenesis of psoriasis through dysregulated keratinocyte proliferation (Fickenscher et al., 2002). In other tissues it has been shown that in addition to this, IL-22 promotes epithelial defense responses through secretion of antimicrobial molecules (Wolk et al., 2004; Wolk and Sabat, 2006; Aujla et al., 2008; Eyerich et al., 2009). Thus, this is a unique and important cytokine with potent effects on tissue repair and remodeling, which has a specific role in the liver in modulating tissue injury.

In man, many Th17 cells can co-secrete IFN-γ, as well as IL-22 (Annunziato et al., 2008; Cosmi et al., 2008). Additionally, recently a subset of cells which secretes IL-22 but not IL-17 or IFN-γ, termed Th22, have been described; however, these have been considered up until now specific for the skin and whether they may play any role in the liver is unknown (Duhen et al., 2009; Eyerich et al., 2009). Overall the role of Type 17 CD4^+^ T cells in the liver during chronic hepatitis C has not been extensively evaluated. A recent study by the group of Rosen, looking at end-stage liver disease, did reveal enrichment of CD4^+^ T cells secreting IL-17 in comparison to blood in 11 patients (Foster et al., 2012), although the nature of the infiltrate at earlier stages of infection remains to be determined, and the co-expression of IL-17 or IL-22 with IFN-γ has yet to be defined.

CD161 is a C type lectin – initially defined as an NK associated molecule – which has been linked to a liver homing phenotype of T cells in health and disease (Northfield et al., 2008; Billerbeck et al., 2010). CD161 is expressed on both CD3^+^CD8^+^ and CD3^+^CD4^+^ T cells (Takahashi et al., 2006) and recently CD161 expression has been linked to both a “T_H_17” and a “T_C_17” phenotype in man (Cosmi et al., 2008; Billerbeck et al., 2010). CD8^+^ T cells expressing high levels of CD161 are highly enriched in the livers of patients with chronic hepatitis, expressing chemokine receptors associated with tissue homing in resting and inflammatory conditions (Billerbeck et al., 2010). These cells have a distinct phenotype and may secrete IL-17, IL-22, TNFα, and/or IFN-γ. Data from the study of cord blood T cells suggest that CD161 expression occurs very early and that only cells that express this molecule, and therefore the master transcription factor RORγt, are able to differentiate further into IL-22 and IL-17 secreting cell populations (Annunziato et al., 2008; Cosmi et al., 2008; Romagnani et al., 2009; Billerbeck et al., 2010; Maggi et al., 2010). Thus, CD161^+^ CD4^+^ T cells possess enhanced potential**to secrete IL-22 compared to CD161 T cells.

Given our prior data on CD8^+^ CD161^+^ T cells discussed above, we therefore characterized in a parallel way the phenotype and function of CD4^+^ T cells in blood of chronically HCV^+^ donors and controls, and analyzed the function of the infiltrate in the liver. Our data suggest that CD4^+^ T cells expressing CD161 are an important component of the inflammatory infiltrate in HCV and other inflammatory liver diseases. This includes a distinct IL-22/IFN-γ^+^ population which is specifically enriched in the liver and may be of particular functional significance.

## MATERIALS AND METHODS

### PATIENTS AND HEALTHY DONORS

Hepatitis C virus infected patients, patients with non-viral hepatitis, and healthy normal donors were enrolled in this study after informed consent: the patient characteristics of the liver biopsy study cohorts are tabulated in **Table 1**. All those studied – both HCV^+^ and non-alcoholic steatohepatitis (NASH) cohorts – were undergoing liver biopsies for diagnostic reasons related to their normal clinical care as in previous analyses (Billerbeck et al., 2010). For healthy donor studies, peripheral blood mononuclear cells (PBMCs) from groups of up to 12 HCV-negative, low risk healthy controls were analyzed. A further set of HCV^+^ patients and intrahepatic lymphocytes (IHL) from explants from five donors undergoing liver transplant were used for the comparative study in **Figure 3** and this information is briefly summarized in the relevant legend. The study protocol conforms to the ethical guidelines of the 1975 Declaration of Helsinki as reflected in a priori approval by the institutions’ human research committees.

**Table 1:** Subject characteristics for *in vitro* analysis.

Subject	Age	Gender	Viral load (U/ml)	Fibrosis (Metavir)
**Chronic HCV**				
18	48	F	1480000	2
19	52	M	1438180	2
20	54	M	1500000	2
21	63	F	2568188	2
22	56	F	938114	2
23	43	M	915735	3
24	28	M	999416	1
25	62	M		1
26	57	M	123277	3
27	50	M	737087	3
28	44	F	572472	1
29	50	M	4011231	2
30	66	F	639838	3
31	72	F	2904622	3
32	49	M	15800000	3
33	65	F	208729	1
34	32	M	112622	1
35	43	M	864679	1
36	33	F	184445	2
**NASH**				
1	29	M	n.a.	
2	53	M	n.a.	
3	32	F	n.a.	
4	56	F	n.a.	
5	55	M	n.a.	
6	40	M	n.a.	
7	54	M	n.a.	
8	51	F	n.a.	
9	39	M	n.a.	
10	53	M	n.a.	
11	32	M	n.a.	

### CELL PREPARATION

Peripheral blood mononuclear cells, liver cells, and liver-derived cell lines were prepared as previously (Spangenberg et al., 2005). Briefly, for the isolation of intrahepatic lymphocytes (IHL) the liver tissue was washed twice in 3 ml of phosphate buffered saline (PBS) containing 1% FCS prior to a careful homogenization through a 70 μm cell strainer (BD Falcon). The homogenized cell suspension was incubated with magnetic microbeads covered with anti-CD4 (Dynal, Oslo, Norway) for 30 min at 4°C. CD4^+^ T cells bound to the microbeads were isolated from the cell suspension by using a magnetic bead concentrator (Dynal). Peripheral CD4^+^ T cells were isolated from 4 × 10^6^ PBMC by the same experimental approach. The purity of each T cell subset was confirmed by FACS analysis and was always >95%. Isolated intrahepatic and peripheral CD4^+^ T cells were resuspended in 1 ml of complete medium and cultured in a 48-well plate (Greiner, Frickenhausen, Germany) in the presence of 100 U/ml human rIL-2 (Hoffmann La Roche, Basel, Switzerland), 0.04 μg/ml human anti-CD3 antibody (Immunotech, Marseilles, France), and 2 × 10^6^ irradiated autologous PBMC as feeder cells. Twice a week the medium was exchanged and 100 U/ml IL-2 was added. After 2 weeks of expansion, cells were analyzed for cytokine production as described below. For *ex vivo* analyses the same approach to isolate the cells was taken to obtain the cells from blood and liver but no *in vitro* selection and expansion steps were used. The cells were stimulated and analyzed in short-term assays as below.

### LYMPHOCYTE STAINING AND ANALYSIS

Lymphocyte staining was performed on whole blood as previously (Northfield et al., 2008), using antibodies as indicated below. Intracellular cytokine staining after PMA/Ionomycin stimulation was performed as previously (Northfield et al., 2008; Bengsch et al., 2012). Cells were analyzed on a BD LSRII machine, BD FACSCanto or BD FACSCalibur machine using FlowJo software. Samples were gated on live, singlet CD3^+^ lymphocytes. Non-parametric tests, Mann–Whitney and Wilcoxon were used throughout for unpaired/paired comparisons respectively (Prism software v5).

### ANTIBODIES

Anti-CD8-PerCP, anti-CD8-PE, anti-CD8-PerCP-Cy5.5, anti-CD8-Alexafuor700-anti-CD4-PerCP, anti-CD4-FITC, anti-CD4-APC, anti-IFN-γ-FITC, anti-TCRγδ-PE, anti-TCRαβ-PE, anti-TNF-α, isotype PE, isotype FITC, and isotype APC were obtained from BD Pharmingen (Heidelberg, Germany). Anti-CD3 FITC/APC, anti-IL-22-APC, anti-CCR6-APC, anti-CCR2-APC, anti-CXCR6-PE, anti-CXCR3-FITC, anti-CCR7-FITC, biotin-anti-IL23R, and anti-IFN-γ-FITC were obtained from R&D Systems (Minneapolis, MN, USA), anti-CD161-APC from Miltenyi Biotech (Bergisch-Gladbach, Germany). Anti-IL17A-PE, anti-IL17A-Alexa647, anti-TCRαβ-APC, anti-CD127-PE, anti-RORγT-PE, and anti-IL18R-FITC were from eBioscience (San Diego, CA, USA). Anti-CD161-PE and anti-CD3-ECD obtained from Immunotech/Beckman Coulter (Fullerton, CA, USA). Anti-CD3-Pacific Orange was obtained from Caltag Laboratories (Buckingham, UK). Anti-RORγT-Alexa 488 was obtained from Cambridge Biosciences (Cambridge, UK). FITC- or APC-conjugated antibodies to CD45RA, CD45RO, CD38, CD62L, CD56, CD103, TGFβRII, PD-1, ICOS, GITR, CD38, CD62L, CCR5, CXCR4, CD85j, CD27, CD28 were obtained from BD Pharmingen and R&D systems. Extravidin-PE was obtained from Sigma (St. Louis, MO, USA).

## RESULTS

### FUNCTION AND PHENOTYPE OF CD161^+^CD4^+^ T CELLS IN HEALTHY DONOR BLOOD

**FIGURE 1:**
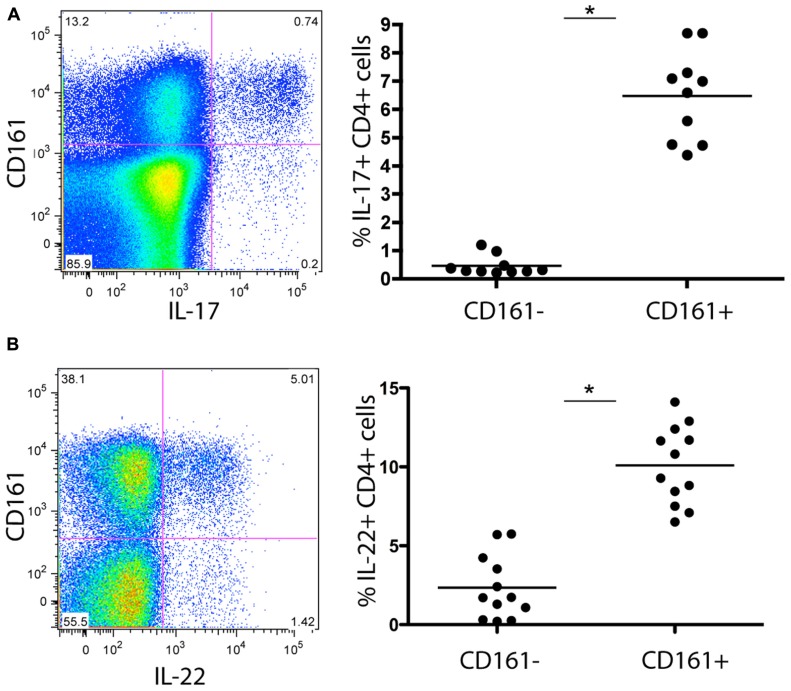
**Cytokine secretion capacity of CD161^+^CD4^+^ T cells in healthy donor blood**. Healthy donor blood *ex vivo* was stained as described in methods and analyzed for expression of the following cytokines following PMA/Ionomycin stimulation: IL-17A **(A)**, IL-22 **(B)** Plots show gated live CD3^+^CD4^+^ lymphocytes. In each case an example is shown on the left and group data on the right, with comparison by Mann Whitney test.

Initially, we examined the immediate effector functions of CD161^+^ and CD161- CD4^+^ T cells in healthy donors. To do this we stimulated populations taken directly *ex vivo* from healthy donors with PMA/Ionomycin and examined cytokine production by intracellular cytokine secretion. CD161^+^CD4^+^ T cells generated high levels of IL-17A upon stimulation, compared to CD161^-^ cells (mean = 7%; **Figure 1A**). We noted co-secretion of IL-17A and IFN-γ, as previously described for CD8^+^ T cells (Billerbeck et al., 2010; data not shown). Importantly, we readily identified *ex vivo* secretion of hepatoprotective IL-22 by a larger fraction of cells, and found this to be strongly associated with the CD161^+^CD4^+^ T cell subset (mean = 10%; **Figure 1B**).

**FIGURE 2:**
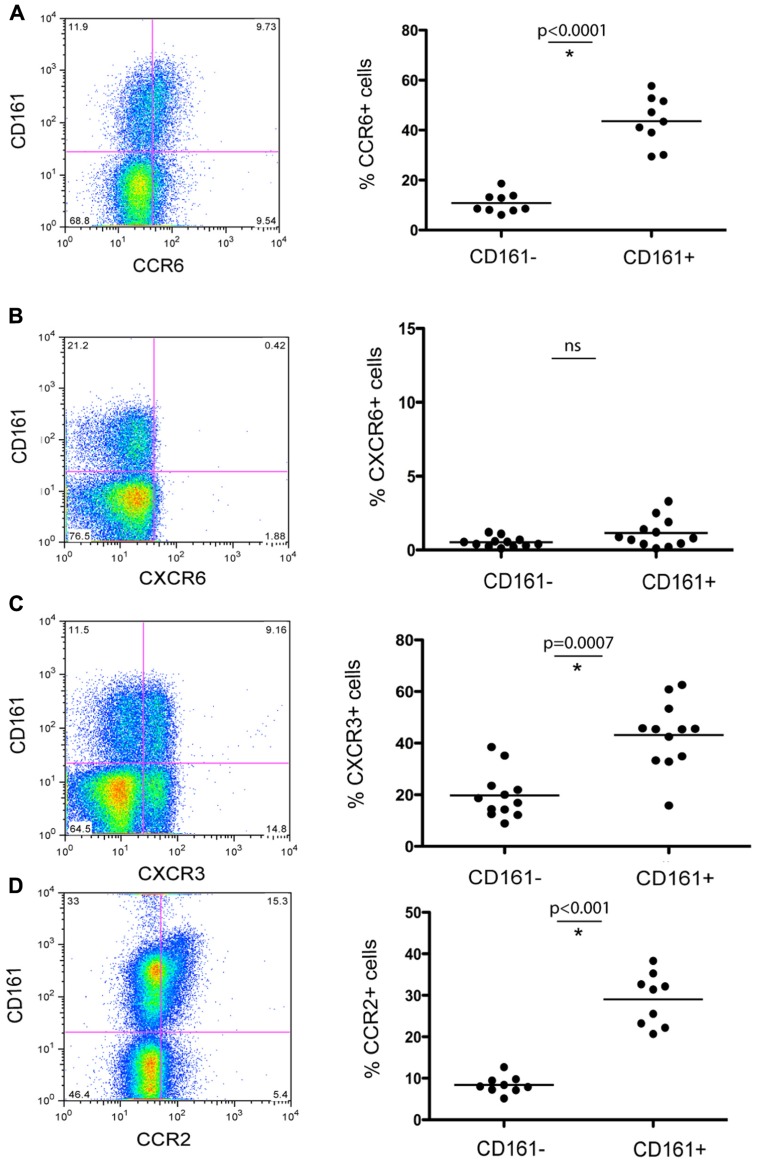
**Chemokine receptor expression of CD161^+^ CD4^+^ T cells in healthy donor blood**. Healthy donor blood was stained as described in methods and analyzed for expression of the following chemokine receptors: CCR6 **(A)**, CXCR6 **(B)**, CXCR3 **(C)**, and CCR2 **(D)**. Plots show gated live CD3^+^CD4^+^ lymphocytes. In each case an example is shown on the left and group data on the right, with comparison by Mann–Whitney test.

To further characterize this subset of T cells for features relevant to liver homing, we next analyzed expression of relevant chemokine receptors in relation to CD161. We found co-expression of CCR6 (45%), as well as CCR2 (30%; **Figures 2A,D**). We noted high expression of CXCR3, a chemokine receptor linked to homing to inflammatory sites (45%) although CD161^+^CD4^+^ T cells did not express CXCR6 – a key chemokine receptor associated with immunosurveillance in the liver sinusoids (Geissmann et al., 2005; Sato et al., 2005; **Figures 2B,C**). We also confirmed that circulating CD4^+^CD161^+^ cells analyzed *ex vivo* expressed the cytokine receptor IL-23R, high levels of the IL-18Rα and increased levels of RORγt expression compared to CD161^-^ cells (data not shown) similar to data from CD8^+^ CD161^+^ cells (Billerbeck et al., 2010). In terms of other effector/memory markers, these CD4^+^ T cells displayed an “effector memory” phenotype (CD45RO^hi^, CD45RA^lo^, CD62L^lo^, CCR7^lo^, CD28^hi^, CD27^hi^, perforin^lo^, Granzyme B^lo^) without evidence of exhaustion (PD-1^lo^; data not shown).

These data confirm and further define some important functional capacities of the CD161^+^ T cell subset. The cells express relevant chemokine receptors for liver homing during inflammation and relevant cytokine receptors and transcription factors for IL17/IL-22 induction and maintenance. The CD161^+^CD4^+^ T cell fraction also includes IL-22^+^ cells with the ability to express IFN-γ and/or IL-17 as well as IL-22 mono-secreting cells.

### CD161^+^CD4^+^ T CELLS ARE ENRICHED IN THE LIVER DURING CHRONIC HEPATITIS C

**FIGURE 3:**
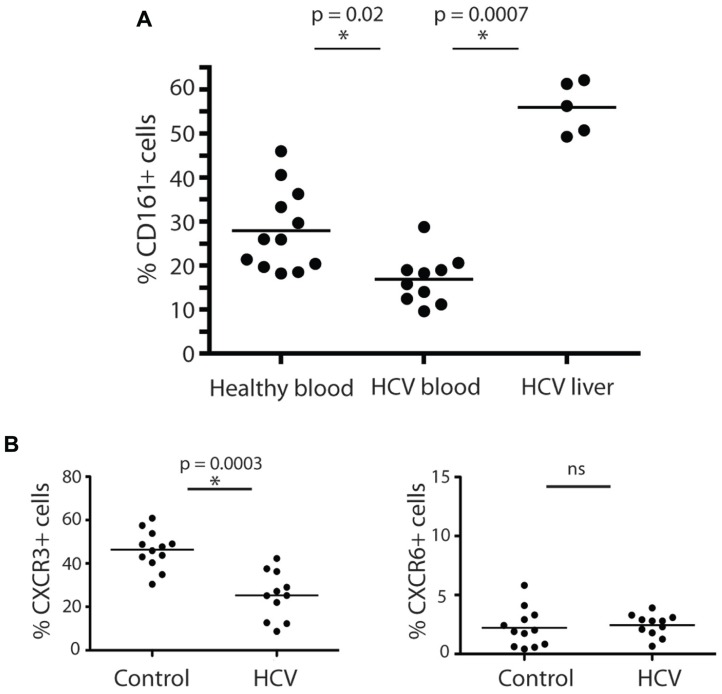
**CD161^+^CD4^+^ T cells in blood and liver in HCV infection**. CD161^+^CD4^+^CD3^+^ T cell frequencies were examined in blood from healthy donors, HCV^+^ chronically infected donors and liver derived lymphocytes, studied *ex vivo* **(A)**. Percentage CD161^+^ cells amongst CD4^+^ T cells in liver explants, PBMC from HCV^+^ donors and PBMC from healthy donors are shown. The 15 HCV^+^ donors studied in the analysis of PBMC were additional to those in **Table 1** and comprised persistently infected donors (eight genotype 1, six genotype 3, one genotype 2), of whom five had Ishak histologic scores of 5^+^/6 and/or clinical cirrhosis; the IHL in this study were obtained at explant for cirrhosis from HCV^+^ donors. Groups were compared by Mann-Whitney tests. In **(B)**, the phenotype of CD161^+^CD4^+^CD3^+^ T cells in peripheral blood of HCV^+^ donors and healthy controls were compared as in **Figures 1 and 2**. Data is shown from CXCR3 and CXCR6 analyses. No significant change was seen in analyses of CD27, CD28, CD45RA/RO, CD62L, CCR6, CD103, or PD-1.

Having established their baseline function and phenotype in healthy donors, we next addressed whether CD161^+^CD4^+^ T cells were associated with intrahepatic infiltrates during chronic hepatitis C infection. We noted a marked enrichment of CD161^+^CD4^+^ T cells derived from liver tissue from HCV^+^ donors, with up to 50% of infiltrating CD4^+^ T cells expressing this molecule (**Figure 3A**). In blood, there was a significant decrease in the frequency of CD4^+^ T cells expressing CD161 in HCV^+^ individuals compared to healthy donors (*p* = 0.02; **Figure 3A**).

We next analyzed whether HCV had any impact on the phenotype of CD161^+^CD4^+^ T cells in blood, in addition to affecting their frequency. We found the relative reduction of CD161^+^CD4^+^ T cells in blood occurred most significantly amongst CXCR3^+^ CD161^+^CD4^+^ T cells (**Figure 3B**). The expression of the other markers on CD4^+^ T cells analyzed, including CXCR6, was not affected by HCV infection (**Figure 3B** and data not shown). This feature is consistent with homing of CXCR3^+^ T cells to the liver as has been indicated in previous studies (Boisvert et al., 2003; Wang et al., 2004).

### ANALYSIS OF CYTOKINE SECRETING CD4^+^ T CELLS IN THE LIVER IN HCV INFECTION

**FIGURE 4:**
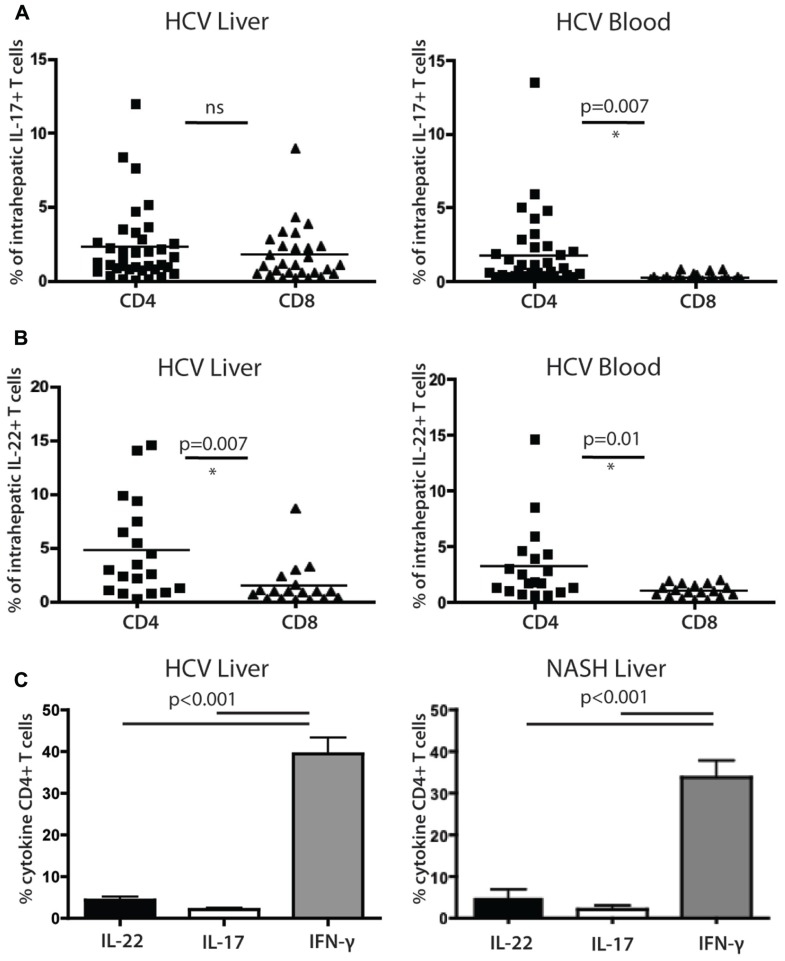
**Analysis of cytokine secreting cells in the liver of HCV^+^ persons and controls**. **(A)** Liver-derived and blood-derived CD4^+^ and CD8^+^ lymphocytes were analyzed for their cytokine secretion capacity as described in methods. The fractions of cells secreting IL-17A are indicated. **(B)** Data for IL-22 secretion in blood and liver are indicated as for IL-17A. **(C)** Analysis of CD4^+^ T cell cytokine secretion (IFN-γ vs IL-22 and Il-17) in liver, in patients with HCV (left panel) and NASH (right panel).

We next examined the functional capacity of CD4^+^ T cells derived from the liver biopsies of HCV^+^ persons, focusing on IL-22 and IL-17 production. Initially, as previously in our studies of CD8^+^ T cells, we used populations of CD4^+^ T cells which had been expanded *in vitro* using non-specific stimuli (Billerbeck et al., 2010). Exactly parallel stimulations with PMA/Ionomycin were then performed using blood and liver-derived cells (**Figures 4A,B**). We compared data between CD4^+^ and CD8^+^ T cell subsets.

We confirmed that IL-22 and IL-17 secreting CD4^+^ T cells were readily detectable within liver-infiltrating lymphocyte (LIL) populations (**Figures 4A,B**). IL-22 secreting CD4^+^ T cells comprised on average 4.7% of the CD4^+^ T cell infiltrate, compared to 2.2% of the IL-17 secreting cells (*p* = 0.02). The levels of CD4^+^ T cells and CD8^+^ T cells secreting IL-17 in liver tissue were not significantly different from each other, while in blood, CD4^+^ T cells secreting IL-17 were significantly more numerous than their CD8^+^ counterparts (**Figure 4A**). For CD8^+^ T cells there was a marked enrichment of IL-17 secreting cells in liver compared to blood, but for CD4^+^ T cells the levels were very similar in both sites and the differences were not significant (**Figure 4A**). IL-22^+^ CD4^+^ populations were also not enriched in liver compared to blood but were significantly more numerous than their CD8^+^ counterparts in both locations (**Figure 4B**). Both IL-22^+^ and IL-17^+^ CD4^+^ T cells were markedly outnumbered by IFN-γ secreting cells (40%; both *p* < 0.001; **Figure 4C**).

To assess whether these results were restricted to HCV, we also compared a set of patients with NASH using identical protocols. Overall, remarkably similar results were obtained (**Figure 4C**); the frequencies and relative distributions of CD4^+^ T cells secreting IL-17, IL-22, and IFN-γ were very similar to those obtained in HCV^+^ patients, with both IL-17^+^ and IL-22^+^ populations present, but outnumbered clearly by IFN-γ^+^ CD4^+^ T cells (*p* < 0.001).

### CO-SECRETION OF IL-22 WITH OTHER CYTOKINES BY CD4^+^ T CELLS IN THE HUMAN LIVER

Since we had identified IL-22- secreting CD4^+^ T cells within the liver infiltrates, we next addressed whether these cells co-secreted other cytokines. Previous reports have described T cells that secrete IL-22 but not IFN-γ or IL-17 (Duhen et al., 2009; Eyerich et al., 2009) in skin (Th22 cells) and we asked whether such cells exist within the liver-homing populations.

**FIGURE 5:**
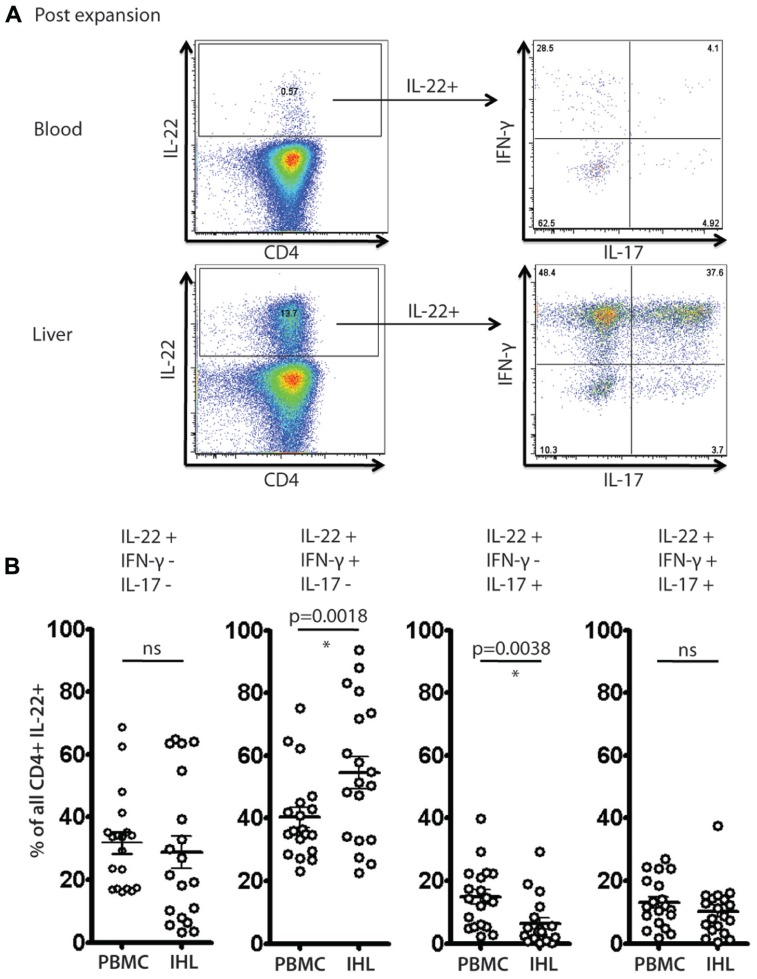
**Analysis of IL-22 secreting cells and polyfunctionality in blood and liver derived lymphocytes**. Liver derived lymphocytes and PBMCs from HCV^+^ donors expanded *in vitro* were analyzed in parallel for expression of cytokines IL-17A, IL-22 and IFN-γ in response to PMA/Ionomycin stimulation as described in methods. In **(A)** an example of staining is shown. IL-22^+^ CD4^+^ T cells were gated upon and co-secretion of IL-17A and IFN-γ was assessed. In **(B)** the fraction of cells secreting IL-22 either alone or in combination with the other cytokines is displayed and compared (paired Student’s *t*-test). A significant enrichment of liver-derived IFN-γ/IL-22 co-secreting cells is observed.

We identified clear subsets of CD4^+^ T cells that have a “Th22” phenotype (**Figure 5A**), i.e., these cells secrete IL-22, but not IFN-γ or IL-17A. However, the frequencies of IL-22 monosecretors as a fraction of IL-22 secreting cells were similar in blood and liver overall (**Figure 5B**). Interestingly, we also noted a consistent and significant enrichment of IL-22/IFN-γ dual secreting cells within the liver compartment (*p* = 0.0018; **Figure 5B**). In the tissue-derived cells these populations represented more than 50% of the IL-22 population on average. Indeed, taking into account those cells that additionally secreted IL-17 (i.e., triple producers; **Figure 5B**), this was clearly the dominant population in the liver.

### ANALYSIS OF *EX VIVO* SECRETION OF IL-22 IN BLOOD AND LIVER DERIVED CD4^+^ T CELL POPULATIONS

**FIGURE 6:**
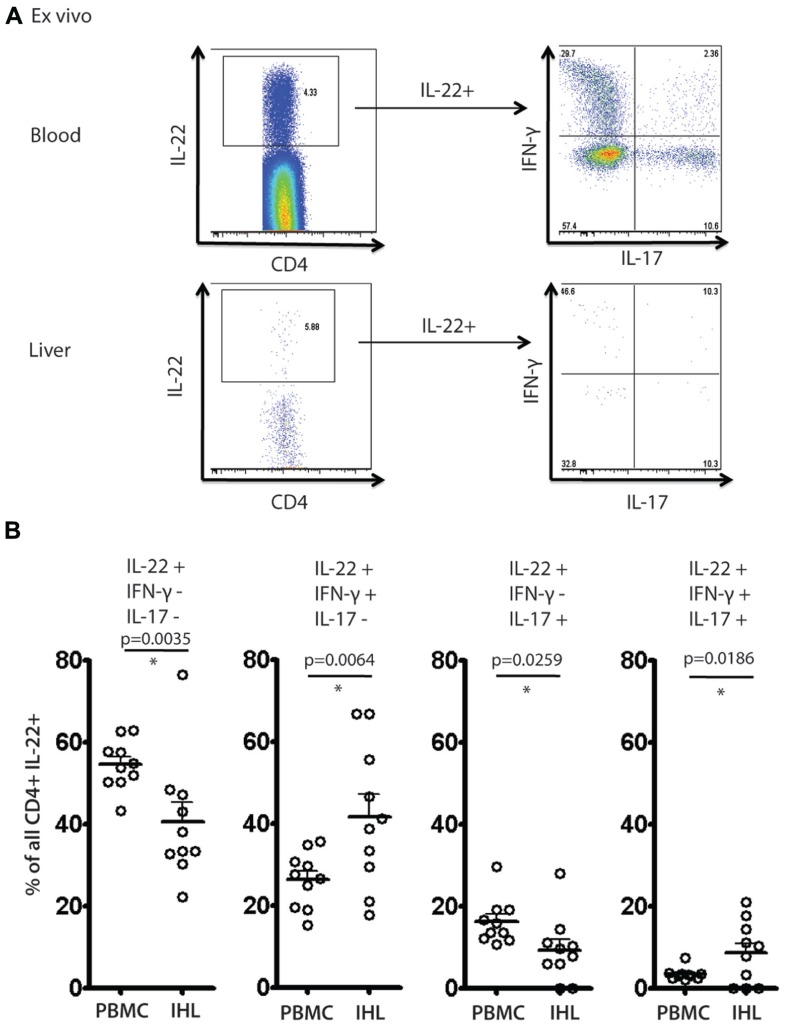
**Analysis of IL-22 secretion and polyfunctionality in blood and liver derived lymphocytes studied directly *ex vivo***. Liver derived lymphocytes and PBMCs were analyzed in parallel directly following isolation for expression of cytokines IL-17A, IL-22 and IFN-γ in response to PMA/Ionomycin stimulation. In **(A)** an example of staining is shown. IL-22^+^ CD4^+^ T cells were gated upon and co-secretion of IL-17A and IFN-γ was assessed. In **(B)** the fraction of cells secreting IL-22 either alone or in combination with the other cytokines is displayed and compared using paired Student’s t test in blood and liver. A significant enrichment of intrahepatic IL-22/IFN-γ co-secreting cells and IL-22/IFN-γ/IL-17A triple secretors is observed.

To address further the significance of these results a further set of analyses were performed *ex vivo* on liver-derived and blood-derived cells from a further 10 patients. *Ex vivo* derived cell populations were those isolated fresh from blood or tissue and then subjected to PMA/Ionomycin stimulation and intracellular cytokine staining without any further expansion *in vitro*. Using these freshly obtained cell populations we observed comparable patterns of response to those obtained with cultured cells. Firstly, we observed readily detectable levels of IL-22^+^ cells *ex vivo* (**Figure 6A**). We next analyzed the polyfunctionality of IL-22^+^ cells regarding co-secretion of IFN-γ and IL-17A. Importantly, once again we found specific enrichment of IL-22/IFN-γ dual secreting cells and also of IL-22/IFN-γ/IL-17A triple secreting cells in the liver, confirming the significance of this finding (**Figure 6B**).

## DISCUSSION

These data provide a novel insight into the composition of the inflammatory infiltrate in chronic liver disease. Despite the clear evidence from animal models, we do not yet know to what extent IL-22 secreting T cells participate in different stages of human liver disease, their impact in tissue, or to what extent measurement of such cells in blood or liver may vary or correlate with clinical outcome. These data represent an important step in the direction of defining the significance of this relevant cytokine in chronic hepatitis.

Although we have focused on HCV, since this is a major clinical problem, a comparable profile was observed in non-viral inflammation. Indeed, since previous data have shown that there is relative enrichment of CD161^+^ T cells in the livers of normal healthy donors (Norris et al., 1999), we postulate this may represent a interesting functional potential of liver homing cells. It should be stressed that the absolute numbers of LILs are relatively low in normal livers, and markedly increased in the setting of chronic liver inflammation. Further work is required to define the cytokine secretion of both the CD4^+^ and CD8^+^ CD161^+^ T cells in the absence of an inflammatory milieu.

We focused on the phenotype and function of CD161^+^ CD4^+^ T cells in health and disease for two reasons. Firstly, CD161 has been linked to LIL populations in humans, as in this paper and in our previous work, which showed enrichment on antigen-specific CD8^+^ T cells specific for hepatotropic viruses (Northfield et al., 2008), as well as on bulk CD4^+^ T cell populations (Foster et al., 2012). Secondly, it has been recently shown that Th17 populations express CD161, using an expression microarray approach Cosmi et al., 2008). Our work is consistent with these findings. CD161^+^CD4^+^ T cells found in blood express not only the classical chemokine receptor associated with Th17 populations, CCR6, but also a range of others, including CCR2 and CXCR3. T cells expressing all three receptors have been found enriched within the liver of HCV^+^ donors (Boisvert et al., 2003; Wang et al., 2004). Functionally, a fraction secrete IL-17A in a short-term stimulation assay, although interestingly a higher frequency in such assays express IL-22, as well as a clear production of IFN-γ. This indicates that there are many IL-22^+^/IL-17^-^ populations, as well as IL-22^+^/IFN-γ^+^ populations, and this was confirmed by dual staining.

The role of the cytokines secreted within liver tissue is an important area to be addressed. IL-17A to date has not been shown to have a major influence on outcome in liver disease models (Zenewicz et al., 2007). In contrast, however, there is a dominant hepatoprotective role of IL-22 *in vivo*. Both blockade of IL-22 and genetic knockout of IL-22 revealed a major effect of this cytokine in the ConA hepatitis model (Zenewicz et al., 2007). IL-22 has a major influence on hepatocyte biology through triggering via the IL-22R. IL-22 may protect directly against hepatic damage since it is known to promote proliferation and limit apoptosis (Wolk et al., 2004; Zenewicz et al., 2007; Dambacher et al., 2008; Park et al., 2010). We have further analyzed the impact of IL-22 on gene expression patterns in human hepatocyte lines *in vitro* and confirm these data, indicating that the major up-regulated gene families relate to cell division (Ramamurthy et al., manuscript in preparation). This is also consistent with the proven impact of IL-22 overexpression *in vivo* in promoting tumor growth (Park et al., 2010).

Recent studies have addressed whether type 17 cells may be identified in the liver during chronic hepatitis B and C (Zhang et al., 2011; Foster et al., 2012), although their overall role in disease progression is still not clear. Foster et al. (2012) addressed the levels of CD4^+^ T cells expressing IL-17 and IL-22 in 11 patients with end-stage liver disease. They noted enrichment of IL-17A, IL-17F, and IL-22 secreting populations in liver compared to blood when analyzed *ex vivo*, although the overall levels of production in liver derived cells were similar to those in this study. An important difference may lie in the nature of the patients, who were undergoing liver transplantation for cirrhosis, compared to the relatively mild disease in our group. We were not able to reliably detect IL-17F by flow cytometry in our studies despite using a range of commercially available antibodies (data not shown).

We attempted to correlate the frequencies of cytokine secreting cells with clinical parameters in our patient groups. Analysis of correlation with disease stage, ALT, or viral load did not reveal any significant relationship for CD4^+^ T cells expressing IL-17, IL-22, or IFN-γ. Although we did not find an association between Th17 or Th22 levels in tissue and clinical state in our study, one caveat is that most patients had relatively mild disease, and there were very few with severe levels of fibrosis. The impact of these cells in long term disease progression (of HCV infection and other inflammatory diseases of the liver), therefore, requires future longitudinal studies.

Interestingly, there do appear to be subsets of “Th22-like” cells in the human liver. This subset has been described by the group of Lanzavecchia; differentiation appears to be driven by IL-6 and TNFα, and may also be triggered by plasmacytoid dendritic cells (Duhen et al., 2009). These cells have been shown to express CCR6, CCR4, and CCR10. CCR6 has been linked to mucosal surveillance and Th17 differentiation (Schutyser et al., 2003; Acosta-Rodriguez et al., 2007; Liu and Rohowsky-Kochan, 2008; Eyerich et al., 2009). Thus, the features of the Th22 subset extend beyond the skin and are likely to include other relevant epithelial tissues and certainly the liver.

The most interesting finding of our study was the consistent enrichment of IL-22/IFN-γ co-producing CD4^+^ T cells in liver. Their *in vivo* function was not elucidated in this study, but interestingly, IL-22R expression may be up-regulated by IFN-γ (Wolk et al., 2004). This implies that the IFN-γ/IL-22 secreting cell population may have a particularly relevant role in this tissue, potentially augmenting the impact of IL-22. IFN-γ itself clearly has important pro-inflammatory and antiviral properties in HCV. Additionally there appear to be many other cytokines and chemokines which may be co-secreted with IFN-γ or IL-22 by the CD161^+^ T cell population (Fleming et al., manuscript in preparation) and, therefore, further description of the true functional potential of the IL-22/IFN-γ-secreting CD4^+^ T cell subset in health and disease is an important future goal.

In summary, we have analyzed the CD4^+^ T cell infiltrate in the liver in chronic HCV, focusing on CD161 expression and associated cytokine secretion. Within liver tissue we identified a population of CD4^+^ T cells which are consistently enriched and which co-secrete IL-22 and IFN-γ. Both these cytokines are of likely significance in the pathogenesis of human liver disease. To what extent these cells play a role in long-term evolution of disease and whether modulation of the appropriate pathways could influence this process, are important questions for the future.

## Conflict of Interest Statement

The authors declare that the research was conducted in the absence of any commercial or financial relationships that could be construed as a potential conflict of interest
